# Successful bilateral nephron-sparing surgery for recurrent stage V Wilms tumor with hepatic capsule involvement: a case report

**DOI:** 10.3389/fped.2026.1772408

**Published:** 2026-02-26

**Authors:** Umer Siddiqui, Meenam Qazi, Sheikh Nabeel Sheikh Mohamed Nazer, Sayed Ayman Ahmed, Youssef Ahmad, Ali Barakat

**Affiliations:** 1 Royal Berkshire NHS Foundation Trust, Reading, United Kingdom; 2 John Radcliffe Hospital, Oxford, United Kingdom; 3 Uniwersytet Medyczny w Lublinie, Lublin, Poland; 4 Burjeel Medical City, Abu Dhabi, United Arab Emirates; 5 University of Tartous, Tartus, Syria

**Keywords:** bilateral wilms tumor, case report, chemoresistance, nephron-sparing surgery, pediatric oncology

## Abstract

**Introduction:**

Wilms tumor is the Most common renal malignancy in children, but bilateral Wilms tumor (BWT) is rare and poses competing priorities for cure and renal preservation. Neoadjuvant chemotherapy followed by nephron-sparing surgery is standard, yet optimal timing when resistance emerges remains challenging. This case describes advanced BWT with secondary chemotherapy resistance and hepatic capsular involvement, successfully managed with timely, aggressive bilateral nephron-sparing surgery and liver capsule resection.

**Case presentation:**

A 5-year-old girl presented with progressive, painless abdominal distension and large, firm bilateral flank masses. Abdominal MRI showed large intrarenal tumors in both kidneys without metastases. After six months of neoadjuvant chemotherapy, initial partial response was followed by interval regrowth. She underwent single-stage bilateral nephron-sparing surgery with right partial nephrectomy plus *en bloc* liver capsule resection and left partial nephrectomy with reconstruction and nephrostomy. The patient is currently disease-free on follow-up.

**Discussion:**

Bilateral Wilms tumor represents a minority of cases and carries a substantial lifetime risk of end-stage renal disease. Protocols favor neoadjuvant chemotherapy to facilitate nephron-sparing surgery within 6–12 weeks. In this patient, secondary chemotherapy resistance with predominant stromal maturation supported proceeding to surgery rather than intensifying therapy. Successful single-stage bilateral nephron-sparing surgery with negative margins avoided dialysis and transplant despite extensive bilateral disease and hepatic capsular extension.

**Conclusion:**

This case demonstrates that even in bilateral Wilms tumor complicated by secondary chemotherapy resistance and hepatic capsular involvement, aggressive nephron-sparing surgery can achieve disease control while preserving renal function. Prolonged oncologic and renal surveillance is warranted given the recognized risk of late renal events.

## Introduction

Wilms tumor, or nephroblastoma, is the most common renal malignancy in children, accounting for approximately 6% of all pediatric cancers (1). While the majority of cases present unilaterally, bilateral Wilms tumor (BWT) occurs in only 5%–10% of patients and represents a distinct clinical challenge requiring an upfront balance between oncological control and renal parenchyma preservation (2). Standard management often involves neoadjuvant chemotherapy—a primary strategy in the International Society of Pediatric Oncology (SIOP) protocols—to facilitate nephron-sparing surgery (NSS). However, the management becomes exponentially more complex in cases exhibiting secondary chemotherapy resistance, interval tumor regrowth, or extensive local extension to adjacent organs (3–5). These atypical presentations demand aggressive and technically demanding surgical solutions to achieve oncological clearance while avoiding end-stage renal failure.

Herein, we report a complex case of stage V BWT characterized by post-chemotherapy regrowth and liver capsule involvement, to highlight the feasibility and success of an aggressive bilateral nephron-sparing surgical approach in the setting of recurrence.

## Case presentation

A 5-year-old girl presented to the clinic with a history of progressive, painless abdominal swelling noticed by her parents over several weeks. No other associated symptoms were reported. On physical examination, she was alert and active, and a large, firm, non-tender, bilateral flank mass was palpated. Abdominal distension was also noted. There was no significant family history of renal disease or malignancy, and the patient had no known prior medical conditions.

**Figure 1:**
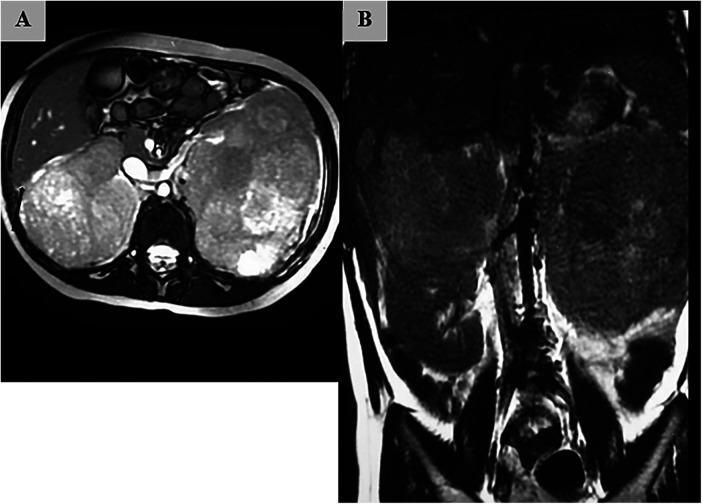
Pre-chemotherapy magnetic resonance imaging (MRI). **(A)** Axial T2-weighted image demonstrating massive bilateral renal masses, with the right-sided tumor showing effacement of the fat plane with the liver (arrow). **(B)** Coronal T2-weighted image showing the extensive size of the bilateral tumors, replacing a significant portion of the renal parenchyma.

Initial diagnostic imaging with abdominal Magnetic Resonance Imaging (MRI) was performed. T2-weighted axial and coronal images revealed massive, heterogeneous, bilateral renal masses, consistent with a diagnosis of Stage V Wilms tumor. The right renal mass appeared to originate from the upper pole, measuring approximately 10 cm × 8 cm, and demonstrated effacement of the fat plane with the posterior surface of the liver, raising strong suspicion for direct adherence to the hepatic capsule (Figure 1A). The left renal mass was similarly large, measuring approximately 12 cm × 9 cm, and appeared to replace the majority of the left renal parenchyma (Figure 1B). No evidence of tumor thrombus within the renal veins or inferior vena cava was identified.

**Figure 2:**
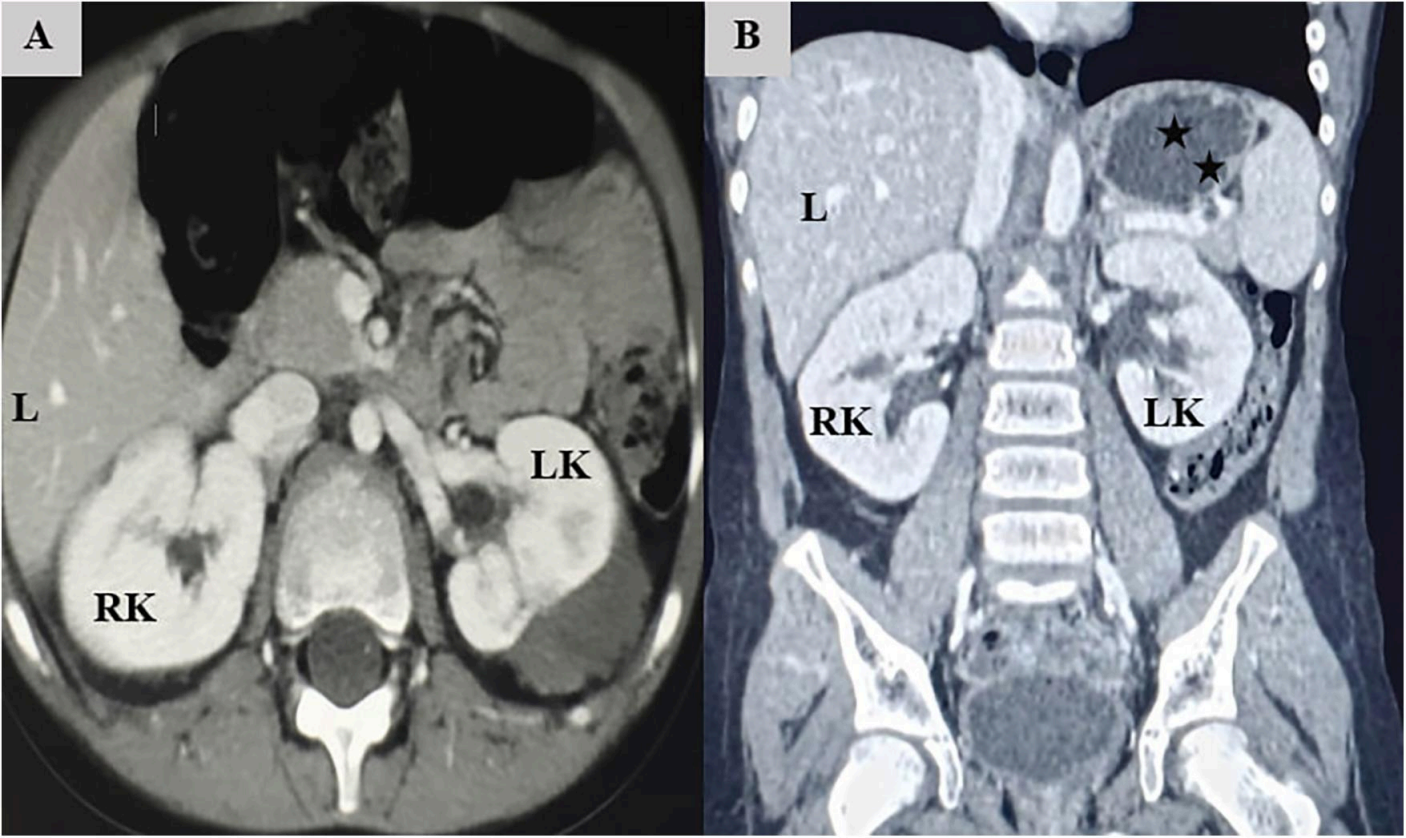
Follow-up computed tomography (CT) after 6 months of neoadjuvant chemotherapy. **(A)** Axial image showing significant reduction in tumor size and restoration of the fat plane between the right kidney (RK) and the liver **(**L**)**. **(B)** Coronal image demonstrating the residual complex cystic mass in the upper pole of the left kidney (LK) (asterisks), and the well-preserved parenchyma of the right kidney (RK).

Based on the highly characteristic radiological findings on the initial MRI and in accordance with the International Society of Paediatric Oncology (SIOP) protocol, neoadjuvant chemotherapy was initiated without a pre-treatment histopathological biopsy. The patient received neoadjuvant chemotherapy for 16 weeks. Due to an initial assessment of insufficient response, the regimen was intensified with high-risk alternating cycles of Doxorubicin (50 mg/m^2^) plus Cyclophosphamide (450 mg/m^2^) and Carboplatin (200 mg/m^2^) plus Etoposide (150 mg/m^2^). This clinical course, combined with socioeconomic barriers to immediate surgical access in our setting, resulted in a total pre-operative period of approximately 6 months. Follow-up contrast-enhanced Computed Tomography (CT) of the abdomen demonstrated a significant partial response. Axial and coronal images showed a dramatic interval decrease in the size of both renal masses, which now appeared smaller and better circumscribed, with large areas of low attenuation consistent with treatment-induced necrosis. Importantly, a clear fat plane was now visible between the right renal mass and the liver (Figures 2A,B).

**Figure 3:**
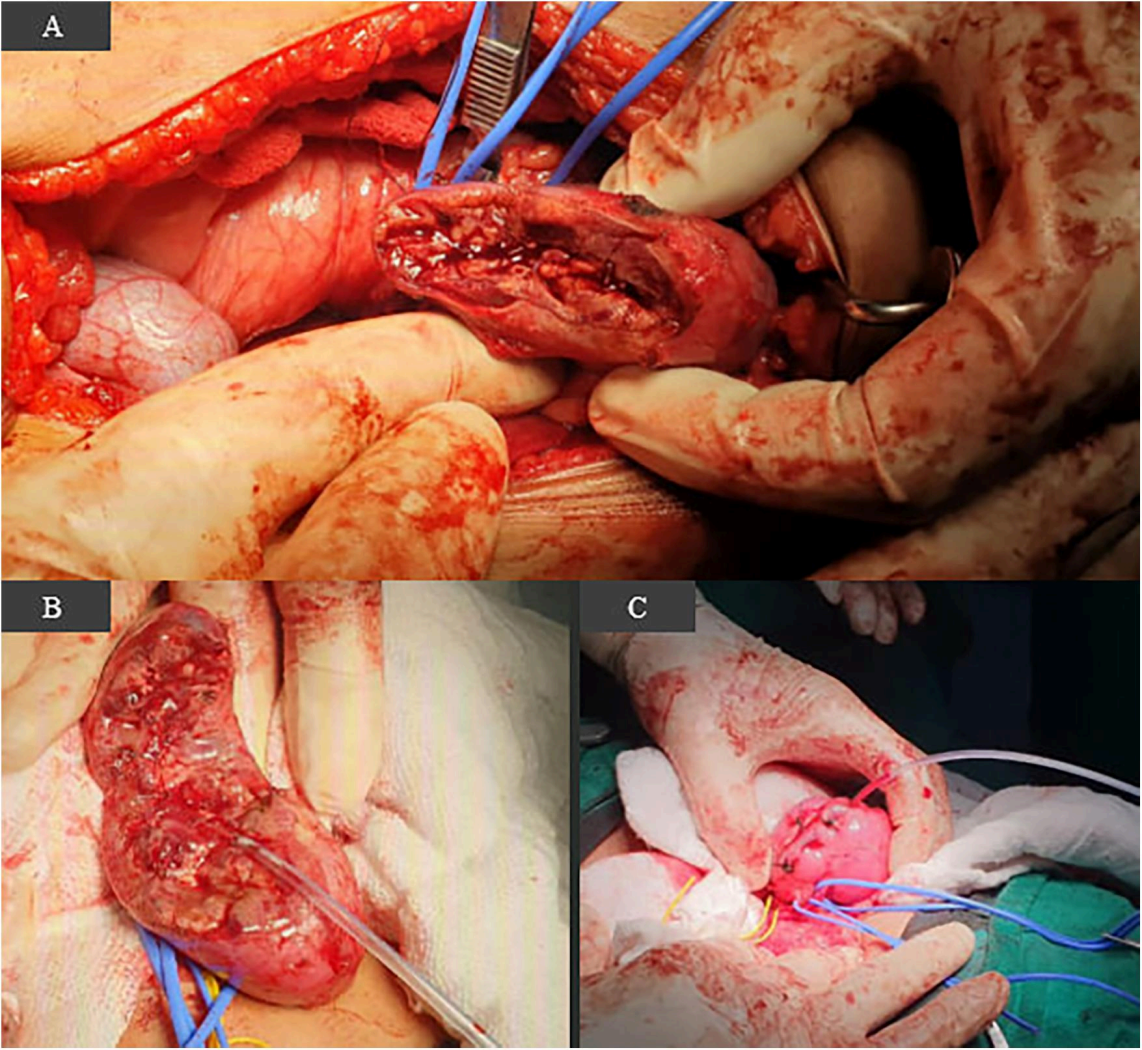
Intraoperative photographs of the bilateral nephron-sparing surgery: **(A)** the right kidney *in situ* after partial nephrectomy of the upper pole tumor. Note the proximity to the liver (retracted superiorly), consistent with the site of capsular adherence. **(B)** The **left kidney** after a 50% partial nephrectomy, showing the resection bed and the placement of a nephrostomy tube. **(C)** The final appearance of the extensively reconstructed and sutured **left kidney** at the conclusion of the procedure.

However, subsequent imaging prior to surgery revealed interval regrowth of the tumors. A multidisciplinary tumor board decision was therefore made to proceed with surgical resection. The patient underwent a single-stage bilateral nephron-sparing surgery, which was performed under warm ischemia to achieve complete gross tumor removal while minimizing total operative and anesthesia time. Intraoperatively, the right upper pole tumor was confirmed to be densely adherent to the liver capsule, requiring an en-bloc resection of the involved portion (Figure 3A). On the left side, a large tumor involving approximately 50% of the kidney was resected, followed by extensive renal reconstruction and placement of a temporary nephrostomy tube, which was removed after approximately 2 weeks following confirmation of adequate urinary drainage (Figure 3B).

Histopathology of the resected specimens confirmed bilateral Wilms tumor with favorable histology and no anaplastic component. According to the SIOP classification, the overall diagnosis was confirmed as Stage V Wilms tumor. The right specimen contained a 4 cm tumor mass staged as **pT3** due to infiltration of the renal capsule. The left specimen revealed a 5 cm mass staged as **pT2**, showing significant therapy effects, including fibrosis, maturation, and prominent striated muscular differentiation. Surgical margins were free of malignancy bilaterally.

The postoperative course was uneventful. Following the surgery, the patient was monitored by the surgical team for a period of 4 months. During this initial follow-up, renal function was objectively confirmed to be well-preserved, with serum creatinine levels consistently remaining below 1 mg/dL. Blood pressure remained within normal limits for her age, and no significant proteinuria was detected. Following the successful surgical recovery, the patient's care was transferred to the oncology department for adjuvant therapy and long-term surveillance.

## Discussion

Bilateral Wilms tumor (BWT) has been recognized as a distinct clinical entity since the early National Wilms Tumor Study (NWTS) series designated it as stage V disease and emphasized the dual goals of cure and renal preservation. NWTS-3 formalized upfront chemotherapy followed by conservative surgery for bilateral cases in the late 1970s (6, 7). Contemporary series confirm that bilateral BWT accounts for only 4%–8% of Wilms tumors and typically presents in the second to third year of life, making this 5-year-old girl older than the average reported age at diagnosis (4, 6–8).

Clinically, most BWT series describe painless abdominal distension with large, firm flank masses as the most common presenting feature, with MRI or CT demonstrating large, heterogeneous intrarenal masses often replacing considerable parenchyma (4, 6, 7). Our patient's presentation with progressive, painless abdominal swelling and massive bilateral renal tumors on MRI, but no vascular thrombus or distant metastases, is therefore highly typical and supports a diagnosis of stage V BWT. The final histology of bilateral favorable Wilms tumor, showing therapy-related fibrosis and muscular maturation, further distinguishes this lesion from other pediatric renal neoplasms that may mimic BWT radiologically—such as nephroblastomatosis or renal lymphoma—and aligns with patterns observed in large bilateral cohorts (1, 2, 7, 8). Integrating these histological findings with the patient's clinical course and classic imaging allowed for a definitive diagnostic confirmation while excluding other differential entities.

Standard management strategies for BWT universally recommend neoadjuvant chemotherapy to shrink tumors, facilitate nephron-sparing surgery (NSS), and reduce the risk of early renal failure. Most protocols aim for definitive surgery within 6–12 weeks (2, 4, 6, 8). The prolongation of chemotherapy in this case beyond the standard 12-week window was necessitated by an initial clinical perception of suboptimal response, prompting the administration of intensified rescue regimens. Additionally, socioeconomic challenges faced by the family in our local environment significantly contributed to the delay in proceeding to definitive bilateral NSS**.** Although Wilms tumor is generally chemosensitive, recent large series show that only about one-third of affected kidneys demonstrate substantial volume reduction and that primary poor response does not necessarily imply anaplastic histology (1, 2, 4). Duncan et al. reported that tumors that increase in size during neoadjuvant chemotherapy are most often stromal-predominant in BWT. They occur in younger children and still have excellent survival, suggesting that escalation or prolongation of chemotherapy is rarely beneficial and that timely NSS is preferable once chemoresistant growth occurs (9). Our patient initially had a marked partial response with decreased tumor bulk and necrosis, followed by interval regrowth before surgery, indicating secondary resistance. In our case, a secondary biopsy at the time of tumor regrowth was deferred. The clinical priority was shifted toward immediate surgical resection to address the evident treatment resistance and to prevent further local progression, as a histological confirmation was deemed unlikely to change the urgent surgical management plan**.** Consequently, the strong stromal and mature elements seen histologically mirror the stromal-predominant, chemotherapy-resistant but prognostically favorable pattern described by Duncan et al., supporting the decision to proceed to surgery rather than further administer chemotherapy (9).

Given BWT is independently associated with a 10%–15% lifetime risk of end-stage renal disease, current consensus advises bilateral NSS whenever feasible. Multiple institutional and cooperative series document that bilateral NSS after neoadjuvant therapy can be achieved in more than half of patients with good event-free survival and preserved renal function (2, 4, 6, 8, 10). Kieran and Davidoff highlight that meticulous bilateral NSS with intraoperative imaging, selective parenchymal resection, and careful collecting system repair yields excellent renal outcomes, and that repeat NSS for local recurrences is feasible without a major increase in renal failure (2, 10). In contrast to many reports in which unilateral radical nephrectomy with contralateral NSS or staged bilateral procedures are used, our patient underwent single-stage bilateral partial nephrectomies with negative margins and extensive reconstruction on the left side, illustrating that aggressive bilateral NSS is achievable even after chemotherapy resistance and can avoid dialysis and transplant (4, 6, 7). Placement of a temporary left nephrostomy for urinary drainage in our patient reflects the general principle, emphasized in nephron-sparing series, that secure urinary diversion with ureteric stents and external drains is crucial after complex collecting-system reconstruction and can support renal recovery (2, 4, 6, 10).

This aggressive surgical approach also reflects the family's perspective; after being fully informed of the technical complexities and potential risks, they prioritized the goal of long-term renal preservation over less conservative alternatives.

Hepatic involvement in Wilms tumor is most commonly reported as true parenchymal liver metastasis. In our case, however, the finding was characterized by dense tumor adherence to the liver capsule with loss of the intervening fat plane on imaging, representing a pattern of local capsular extension rather than frank parenchymal metastasis. While these are distinct clinical entities, we utilized the literature on surgical resection of liver metastases by analogy, as a recent systematic review suggests that aggressive surgical clearance in cases of hepatic involvement—particularly when complete—is associated with better overall survival (5). This anatomy allowed complete oncologic clearance while preserving hepatic parenchyma and is consistent with data showing that complete surgical resection of liver metastases is associated with improved overall survival and low reported rates of major postoperative complications (5).

Long-term cooperative data, including the AREN0534 follow-up and recent prognostic reviews, report 4- to 8-year event-free survival of around 75%–82% for BWT. They also highlight a non-trivial risk of late, often renal-site events that may represent metachronous primaries in predisposed kidneys rather than classical relapse. Given our patient's favorable histology, absence of metastatic disease, successful bilateral nephron-sparing surgery with clear margins, and current disease-free status, she appears representative of the lower-risk subgroup within modern bilateral Wilms tumor cohorts, while still subject to the documented risk of late renal events (1, 5, 8).

## Conclusion

This case illustrates that bilateral Wilms tumor with secondary resistance to chemotherapy can be managed safely and effectively with a timely and aggressive nephron-sparing surgical approach. Our experience, which involved achieving single-stage bilateral partial nephrectomies with clear margins and *en bloc* resection of the involved liver capsule, demonstrates that long-term cure with renal preservation is an achievable outcome, reinforcing the principle of prioritizing nephron-sparing techniques in BWT whenever feasible.

## Data Availability

The original contributions presented in the study are included in the article/Supplementary Material, further inquiries can be directed to the corresponding authors.
